# Analysis of Gene Regulatory Networks of Maize in Response to Nitrogen

**DOI:** 10.3390/genes9030151

**Published:** 2018-03-08

**Authors:** Lu Jiang, Graham Ball, Charlie Hodgman, Anne Coules, Han Zhao, Chungui Lu

**Affiliations:** 1 Provincial Key Laboratory of Agrobiology, Institute of Industrial Crops, Jiangsu Academy of Agricultural Sciences, Nanjing 20014, China; jldeer26@163.com; 2 The John van Geest Cancer Research Centre, Nottingham Trent University, Nottingham NG11 8NS, UK; graham.ball@ntu.ac.uk; 3 University of Nottingham, Sutton Bonington Campus, Loughborough LE12 5RD, UK; charlie.hodgman@nottingham.ac.uk; 4 School of Animal Rural & Environmental Sciences, Nottingham Trent University, Nottingham NG25 0QF, UK; anne.coules@ntu.ac.uk; 5 Provincial Key Laboratory of Agrobiology, Institute of Agricultural Biotechnology, Jiangsu Academy of Agricultural Sciences, Nanjing 20014, China

**Keywords:** maize, nitrogen response genes, regulatory network inference, network analysis

## Abstract

Nitrogen (N) fertilizer has a major influence on the yield and quality. Understanding and optimising the response of crop plants to nitrogen fertilizer usage is of central importance in enhancing food security and agricultural sustainability. In this study, the analysis of gene regulatory networks reveals multiple genes and biological processes in response to N. Two microarray studies have been used to infer components of the nitrogen-response network. Since they used different array technologies, a map linking the two probe sets to the maize B73 reference genome has been generated to allow comparison. Putative *Arabidopsis* homologues of maize genes were used to query the Biological General Repository for Interaction Datasets (BioGRID) network, which yielded the potential involvement of three transcription factors (TFs) (GLK5, MADS64 and bZIP108) and a Calcium-dependent protein kinase. An Artificial Neural Network was used to identify influential genes and retrieved bZIP108 and WRKY36 as significant TFs in both microarray studies, along with genes for Asparagine Synthetase, a dual-specific protein kinase and a protein phosphatase. The output from one study also suggested roles for microRNA (miRNA) 399b and Nin-like Protein 15 (NLP15). Co-expression-network analysis of TFs with closely related profiles to known Nitrate-responsive genes identified GLK5, GLK8 and NLP15 as candidate regulators of genes repressed under low Nitrogen conditions, while bZIP108 might play a role in gene activation.

## 1. Introduction

Maize (*Zea mays* L.) is one of the most important cereal crops in the world and requires high nitrogen (N) fertilizer input. The majority of maize yield increase has been achieved by the use of large quantities of inorganic fertilizer. Consequently, this has caused many problems for both intensive arable farming and the environment [1]. To avoid nitrogen pollution and to maintain a sufficient profit margin, farmers and breeders have to reduce the use of nitrogen fertilizer and employ plant varieties that have better nitrogen use efficiency (NUE). Currently, only 30–50% of applied nitrogen fertilizer is taken up by crops [2]. It is crucial to improve NUE by increasing the ability of crops to uptake, assimilate nitrogen. 

NUE is often expressed as fresh weight or yield per unit of available N in the soil. It can be calculated as the ratio between the amount of fertilizer N removed with the crop and the amount of fertilizer N applied. NUE in plants is a complex phenomenon that depends on several internal and external factors, which include soil nitrogen availability, uptake and assimilation, photosynthetic carbon, nitrate signalling and regulation. Moreover, sustained decreases in fertilizer input and improved or stabilized yield require an improved understanding of NUE. Two major approaches may be taken to understand NUE. Firstly, the response of plants to N deficiency stress can be explored to identify processes affected by such stress. A second approach makes use of natural or induced genetic variation. Therefore, an increased understanding of the mechanisms controlling plant growth and development under N stress conditions is essential for improving NUE and for reducing excessive input of fertilizers. Previous studies have shown transcriptome profiling can provide information about gene expression associated with NUE and highlight important genes. In addition, transcriptome profiling with gene regulatory network has the potential to be used to integrate information on metabolic processes, which include pathways involved in N uptake, N-assimilation and remobilization. Recent advances in plant molecular biotechnology, combined with dynamic integrative biological studies, have expanded our understanding of the regulatory mechanisms controlling the primary steps of inorganic N assimilation and the subsequent biochemical pathways involved in N supply for secondary metabolism. Numerous N-related genetic experiments have been undertaken in *Arabidopsis* and the main crops—rice, wheat and maize. The first maize NUE related traits, quantitative trait loci (QTLs), were reported in 1999 [3]. Later research has shown coincidences between QTLs and traits related to NUE that are controlled by specific genes involved in nitrogen metabolism, N-uptake and N-remobilization [4,5]. 

In the last 15 years, transcriptomics has been widely applied to identify nitrogen-regulated genes and to characterize responses to nitrogen availability. Several classes of N-responsive genes have been identified from microarray studies in *Arabidopsis,* including known nitrate-induced genes (NR genes), the nitrate transporter (NRT1), glutamine synthetase (GS) and additionally many novel genes differentially expressed under low- and high-N conditions [6,7,8]. Many other groups have also investigated the roles of genes (e.g., transcription factors, kinases and nitrogen transporters) related to the regulation of N assimilation by whole genome/transcriptome and transgenic approaches. For example, over-expressed *OsENOD93-1* (N-responsive transcription factors) could improve NUE in rice [9]. Recently, heterotrimeric G proteins that regulate nitrogen-use efficiency were reported in rice [10]; similar work has been carried out in *Arabidopsis* [11] and maize [12] with *Dof1* (DNA-binding with one finger). The transgenic plants with over-expressed *Dof1* showed increased plant growth, photosynthesis and N-assimilation under low N condition. The transcription factor NLP7 [13], PHR1 [14] and protein kinase AtCIP8 [15] were shown to play a key role in nitrogen sensing and signalling in *Arabidopsis*. More recently, micro RNAs (miRNAs) associated with nitrate response have been investigated [16]. miR393/AFB3 has been defined as a unique N-responsive module that controls root system architecture in response to external and internal N availability in *Arabidopsis* [17]. Genes mediated by miRNA, in maize, could have potential use for NUE improvement [18]. 

Many transcriptional profiling studies on NUE have focused on *Arabidopsis* and rice, but, in the last 2–3 years, an increasing number of whole genome transcriptome analyses have been conducted in maize [19,20,21,22,23]. Recently, two groups [24,25] identified the core set of consistently nitrogen-responsive genes by transcriptome approaches with different methods Affymetrix array and Agilent array respectively, in different maize lines. Their initial results showed that several genes were related to a change in N conditions, but their level of response appeared to be largely dependent on the genotype. These genes are involved in a variety of developmental, metabolic and regulatory functions, such as transcription factors that are responsible for coordinating multiple genes and a potential gene regulatory network. Interestingly, a small set of N–responsive genes could be used as biomarkers for both NUE breeding and optimizing fertilizer usage [24]. N starvation could affect genes that are involved in nitrate reduction and amino acid assimilation, ammonium assimilation, carbon assimilation and a slight accumulation of starch. The decrease in N availability also resulted in accumulation of phosphate in the leaves [25]. 

Large-scale gene expression studies by transcriptomic analysis have identified many genes that are associated with N metabolism. However, the absolute level of expression of these genes cannot be determined directly for the coordinated regulation. Some genes are regulated by a single input mechanism; however, in higher organisms, a gene often responds to multiple signals via the activity of diverse transcription factors. Gene interaction studies can give critical insight into the regulation of the N response and NUE. Initiating a program of increased or decreased gene transcription level in turn allows for further coordinated changes and activities, such as recruitment of cofactors, cooperation with other transcription factors, needed for N responses. While we know a lot about the function of individual transcription factors, there is little information about the system as a whole. 

Advanced network analysis allows identification of potential regulators and understanding of the complex interactions taking place. These networks consist of nodes (usually the genes) and edges (the interaction between them). They can be represented as undirected graphs (whose edges might correspond to correlated and anti-correlated relationships), Boolean networks (in which every edge corresponds to an activation/repression switch), or a directed graph with weighted edges (where the +/− sign and weight, respectively, correspond to activation/repression and a measure of the confidence of the interaction, usually as a Bayesian posterior probability). The edges in these networks might be derived from known interaction and regulatory data, but can also be inferred using a broad range of algorithms [26].

The aim of this investigation was to increase the understanding of plant responses to low N inputs with a view to improving NUE. The methods for identifying target genes from transcriptomic data are valuable approaches by providing a general framework for computational modelling of inferred networks. First, it provides proof of principle for a new protocol and algorithm that make it possible to validate microarray data. Second, it is worth using network inference techniques to identify key genes, transcription factors (TFs) specific to NUE. 

## 2. Material and Methods

### 2.1. Microarray Data

Two microarray datasets were used, which were downloaded from NCBI Gene Expression Omnibus with accession numbers GSE32361 and GSE40678. Study 1 contains 90 Affymetrix arrays (Affymetrix Inc., Santa Clara, CA, USA) from 4 maize lines (Line 1–4) at the V6 developmental stage (21 days for sufficient nitrogen treatment and 28 days for limiting nitrogen treatment) with 3 N treatments sufficient, limiting and recovery from limiting) and 3 time points [24]. For consistency, daytime point at 10:00 a.m. was used. The Study 2 dataset holds values for 32 Agilent arrays (Agilent Technologies Inc., Santa Clara, CA, USA), including two maize inbred lines A188 and B73, two treatments (high N and low N) and two time-courses (20 and 30 days after germination [25] (Table 1). All array data were normalized using GeneSpring GX v11.5 (Agilent Technologies Inc.). 

There were 112 genes found from Study 1 whose expression levels were consistent with N status regardless of genotype or other environmental factors and this core set was used for further study. Study 2 might shed more light on genes conferring an advantage tolerating low N levels. In particular, it could be expected to see a stronger response because the N levels in the low N condition are over 10 fold lower. Data from Study 2 should only be compared to the morning samples from Study 1. In terms of their growth, A188 and B73 both tolerated low N to a similar degree, but their response mechanisms appear to be different. At the transcript level, 46%, 26% and 12.4% of variance was respectively due to the genotype, growth stage (leaf 5 versus leaf 6) and N treatment. At the metabolite level, N treatment accounted for more variation than genotype (39.5% versus 24.8% of variance). Given the major transcriptional differences in relation to growth stage, the Day 20–leaf 5 data should not be used in comparisons with Study 1. From both studies, we might, therefore be able to find out parts of the core N response network but also find out which transcript levels alter more strongly in response to different low N conditions. Potential TFs or other regulators may also be identified.

### 2.2. Differential Expression Analysis and Comparison

Two microarray experiments used different chip technologies, which lack common identifiers for comparison. All the probe sets in each chip were mapped to the B73 genome (RefGen v2 5b.60) [25] by sequence alignment. The probes with unique blast hits were retained and obtained a corresponding gene identifier. The 84,246 Study 1 probe sets retrieved 52,304 hits representing 31,958 unique transcripts; while Study 2 with 41,838 probe sets had 35,086 hits representing 27,278 unique transcripts. There were 20,121 unique overlap genes across the two chips. Transcriptional expression profiles under limiting and sufficient nitrogen treatments at the day time were selected, and analysed under low versus sufficient N by National Center for Biotechnology Information Gene Expression Omnibus (NCBI GEO) microarray analysis tool GEO2R each chip separately [27]. A *t*-test was used to select differential expressed probe sets with at least ±2-fold mean difference between sufficient and limiting nitrogen treatments at *p* ≤ 0.05. Nitrogen response genes were integrated across chips comparison via B73 common identifier.

### 2.3. The Steps for Identifying Potential Regulators

The identification of potential regulators of a set of differentially expressed (DE) genes followed three steps. The first involved mapping the genes onto known networks, specifically, Biological General Repository for Interaction Datasets (BioGRID) [28] and Ingenuity Pathway Analysis (IPA, QIAGEN, Redwood City, CA, USA); the second involved inferring networks from DE gene profile data, preferably using Ensembl techniques to focus on the most likely node-edge combinations. Although the detailed topology of such networks may be spurious, the ranking of genes using graph theoretic metrics (e.g., vertex degree, betweenness or closeness) is an unbiased approach more likely to reveal novel genes involved in the process [29]. The final step involved looking for potential regulators in subgraphs from inferred networks that connect DE genes of interest. 

### 2.4. Mapping Differentially Expressed Genes onto Known Networks

The Ensembl Genome BioMart provided an access to annotated expression microarray probes across taxonomic space [30]. *Zea mays* genes AGPv3(5b) were mapped to *Arabidopsis* genome using EnsemblPlants [31] “BioMart v0.7” to build up a Maize-*Arabidopsis*-Map. The 112 DE core genes in Study 1 were mapped to “Maize-*Arabidopsis*-Map” in Table S2. The resulting *Arabidopsis* gene IDs were used for retrieving the sub-network from BioGRID *Arabidopsis* database [28]. The retrieved interactions were imported into Cytoscape v2.8.2 [32] for further analysis and display. The set of *Arabidopsis* ID were then mapped to their Affymetrix probe sets, which were then used as a query set in IPA.

### 2.5. Artificial Neural Network Inference

A multi-layer feed-forward back-propagation of Artificial neural network (ANN) [29] method was applied to the two sets of maize N-response microarray data. Two approaches were used to build up N response networks: one was focused on selected transcription factors of each set, while the other one was a totally blind analysis applied to entire expression datasets. ANN does not show good performance with high dimensional data, so optimized stepwise approaches were used by reducing the marker numbers to a small size. This method included two main stages: screening the Top 100 genes and network building up [33].

The first stage concerned screening and ranking the Top 100 genes. The basic algorithm of this ANN first considered each probe as single inputs, and consequently built none-input models. A cross-validation strategy, combined with an early-stopping method, was applied to avoid over fitting the model to the training data. At each one-input a sub-model was applied by randomly assigning the samples of the dataset to a training test, or validation set. This process was repeated 50 times, for which a complete random reshuffling of the samples in the three subsets was undertaken. After training, all the genes were tested through this method, and all the genes were ranked by their mean square error at predicting phenotype.

Second stage concerned network inference. The ANN was used in such a way that the Top 100 ranked genes were applied as the input to predict the output. The models were then parameterized to provide a weight and sign (i.e., activation or repression) to the mutual interactions between each of the 100 genes and the output ones. All 99 interaction models were then integrated to infer a network. If all interactions were considered, then 9900 potential mutation interactions would be present in the Top 100 genes. For ease of interpretation, only the Top 200 (2%) of interactions were mapped, indicating the strongest potential gene to gene associations. Network analysis and display were implemented by Cytoscape v2.8.2.

### 2.6. Mutual Information Network Inference 

The DeGNServer [34] was used to infer networks for both the Affymetrix and Agilent datasets. Networks with reduced average vertex degree were generated based on co-expression (cut-off > 0.8) and Context Likelihood or Relatedness (CLR, at a cut-off of > 3.6). These networks were downloaded into Cytoscape to rank genes. Common subgraphs of key DE genes were produced in both the DeGNserver and Cytoscape.

### 2.7. Plant Materials

Maize inbred lines B73, B97 were used for RNA extraction. Three days after germination, seedlings were transferred into pots of 1.5 L volume containing none N peat soil. Fertilization started at Day 7 after germination with a modified Hoagland solution (5 mM CaCl_2_, 2 mM MgSO_4_, 2 mg/L Fe, 0.5 mM KH_2_PO_4_, 50 μM H_3_BO_4_, 10 μM MnCl_2_, 1 μM ZnSO_4_, 0.3 μM CuSO_4_ and 0.5 μM Na_2_MoO_4_). Gradient nitrate nutrient solution was applied, 15 mM for sufficient N condition and 0.15 mM KNO_3_ for low N condition. The plants were supplied with 100 mL of nutrient solution every third day, between the fertilization supply, and plants were watered with distilled water depending on water status in the pot. The growth conditions in the growth room were set at diurnal rhythm of 14 h of light at 28 °C with 75% of humidity, and 10 h of night at 20 °C with 50% of humidity. 

### 2.8. RNA Extraction

Whole leaf samples for RNA extraction were collected at 30 days after the start of the germination. They were homogenized with cooled mortar and pestle and aliquoted with liquid nitrogen. Total RNA was extracted with RNasey Plant Mini Kit (QIAGEN, Hilden, Germany), and RNA purification was performed using RNase-Free DNase Set (QIAGEN, Hilden, German).

### 2.9. qRT-PCR Analysis

To validate candidate genes selected from network analysis, expression levels were determined by quantitative RT-PCR. Specific primers were designed to generate PCR products of about 150–250 bp, using the cDNA sequence information [25]. B73 and B97 leaf samples under sufficient nitrate 15 mM and low nitrate 0.15 mM treatment were used for qRT-PCR analysis. Reverse transcription reactions were performed using 1 μg of total RNA and the Superscript III Reverse Transcriptase (Invitrogen, Carlsbad, CA, USA). The reactions were performed using a LightCycler 480 (Lifescience USA, Zurich, Switzerland), with a final volume of 12 μL PerfeCTa SYBR Green kit (Quanta bioscience, Beverly, MA, USA). The qRT-PCR profile used: 95 °C for 30 s; 40 cycles of 95 °C for 10 s, 56 °C for 10 s and 72 °C for 10 s. N treatment specific housekeeping gene UPF 1 was used as a control. The overall standard error of the mean normalized expression was obtained by applying the error calculation based on Taylor’s series as developed for REST software [35]. GS2, NR, and nitrite reductase (NIR) were chosen as N status genes, while ZmASN4, ZmGLK5, and ZmNLP15 picked up from network analysis were down-regulated under low N condition.

## 3. Results

### 3.1. Comparison of Differentially Expressed N-Responsive Genes across Two Microarray Datasets

In this study, two different microarray datasets were compared and analysed. They contained gene expression either at sufficient N or low N with six maize lines in leaf tissue. There were 165 genes in common to both microarray platforms (Table 2 and Table S1). Detailed information for the datasets is shown in Table 2 and Table 3 and also in the Methods Section. Firstly, DE genes, listed in Table 3 for both datasets, were identified. Table 3 shows that different maize lines had different responses to low N. Lines 1 and 3 had 1.5 times more up-regulated than down-regulated genes, while Line 2 and 4 and A188 had more down-regulated than up-regulated genes (Table 3). This suggested that maize lines adopt one of two different gene expression strategies in response to low-N conditions.

**Figure 1:**
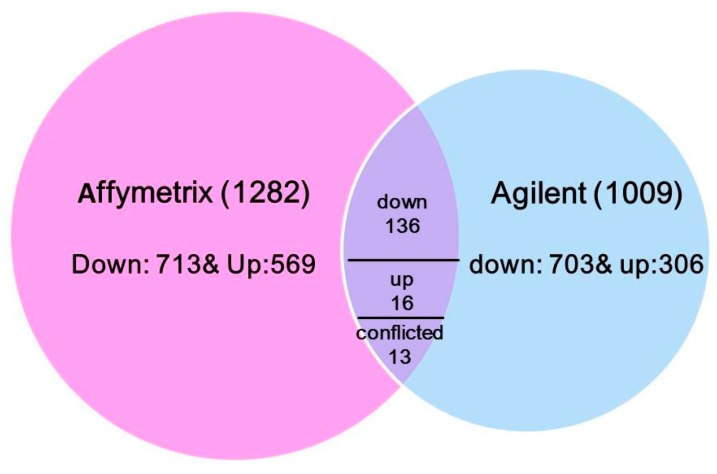
A Venn diagram of differential expressed genes and their common genes across two microarray datasets.

To understand the transcriptional response to N treatments across varieties diversity, the common DE genes from six maize lines (Lines 1–4, A188 and B73) were integrated via the maize reference genome B73 gene identifiers. In total, 165 common genes were identified: 136 genes were down-regulated, 16 genes were up-regulated in all six lines, and 13 genes had conflicted effects under low N vs sufficient N condition (Figure 1 and Table S1). Out of all conflicted probe sets, six were based on the same transcript but located different gene regions (transcription termination sites (TTS), untranslated region (UTR), intron or exon) in two microarray datasets, partially explaining the discrepancy between the two experiments.

**Figure 2:**
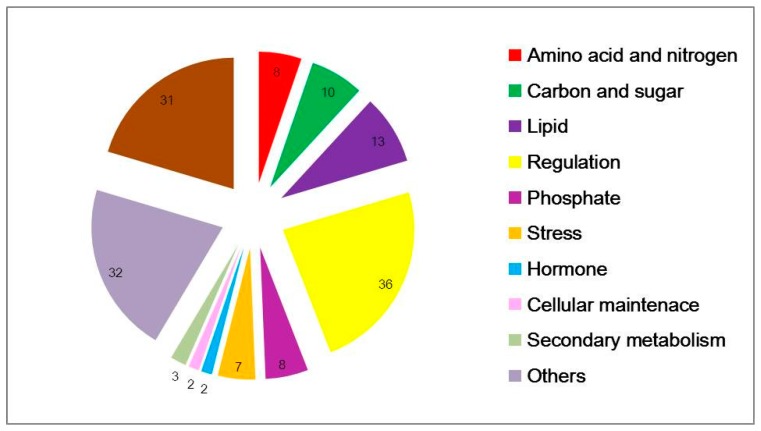
Functional categorization of differential expressed genes in common between two microarray datasets. The probe set sequences were mapped to the UniRef protein database and then subsequently to KEGG function categories, as described in the text. KEGG: Kyoto Encyclopedia of Genes and Genomes.

According to probe functional descriptions, 152 common concordant transcripts were categorized into 11 subgroups (Figure 2). Under low-N conditions, major cellular exchange activities and anabolism were impeded. The most common amino-acid, nitrogen, carbon-related genes and all of lipid, phosphate and stress-related genes were down-regulated. Amino acids such as asparagine, arginine, serine, threonine were the most strikingly affected, and 13 lipid-related genes were differentially expressed which were associated with intercellular and intracellular molecular transport and exchange. Asparagine is a crucial compound for N transport and storage. Genes containing SPX domains, related to phosphate transport and response to phosphate starvation, were strongly down regulated, underlining the great importance of phosphate homeostasis under nitrogen stress [25]. There are 36 potentially regulatory genes, including protein kinases, phosphatases and 12 TFs, particularly CCAAT-HAP2, CCCH (C3H) zing-finger, ethylene-responsive element binding protein (EBERP), Golden2-like (GLK), Nodule inception protein-like protein (NLP), NF-YC family and MYB (Table 4 and Table S1).

### 3.2. Subnetworks from Known Networks

**Figure 3:**
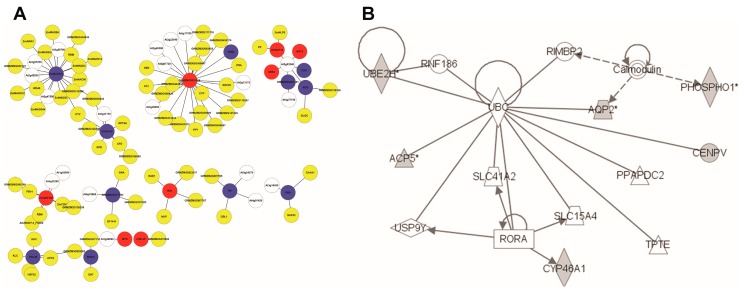
The known network of maize N response genes from Study 1. (**A**) The Biological General Repository for Interaction Datasets (BioGRID) network with maize N response genes: 108 genes with 97 interactions retrieved. Nodes in white represent genes only mapped in *Arabidopsis*; nodes in yellow represent genes with no expression difference; nodes in blue represent down-regulated genes; and nodes in red represent up-regulated genes. (**B**) The IPA network with N response genes. The grey nodes are from the input set of nine genes.

The Study 1 core set of 112 N response gene [24] biomarkers were selected for known network analysis. Ninety-eight of these Affymetrix IDs were associated with 89 maize gene IDs. Only 35 of these were significantly differentially expressed in Study 2, perhaps reflecting the difference in array technology. This small number pulled almost nothing of note out of BioGRID and IPA, and was not investigated further. Searching the Ensembl Maize-*Arabidopsis* homology map found that 80 of the 89 genes mapped to 108 *Arabidopsis* genes (Table S2). The list of *Arabidopsis* genes resulted in the set of 97 interactions from BioGRID is found in the Supplementary Materials (Table S3). This file was imported directly into Cytoscape giving the network (Figure 3A).

The largest component consists of 28 nodes, many of which are transcription (related) factors. Most significantly, the node with the largest number of potential protein interactions encodes ZmMADS76. This is an interesting fact because MADS-box TFs form dimers and often tetramers that recruit enzymes associated with epigenetic gene regulation through histone modification and DNA methylation. The *Arabidopsis* homologue of ZmMADS76 [36] also binds to members of the WD40 family [37], which act as an intermediary protein in heterotrimers. In this case, through Topless and Topless-related-2, it binds to a MYB-like TF homologous to ZmGLK5 [38], indicating a potential co-regulatory role. The expression of both these maize genes reduces in low N, supporting the idea that they might work together. From the Yang core set, another TF (ZmbZIP108) was also retrieved [39], and this potentially binds to a C2H3 TF known to be involved in nitrate transport in *Arabidopsis*, an RNA binding protein, a subunit of the RNA polymerase complex and glutaredoxin. 

The repression of the ZmPK gene might result in less phosphorylation of four members of the calcineurin family, which a calcium-sensors affecting the activity of Ca-dependent protein kinases. The third largest cluster has the *Arabidopsis* poly-ubiquitin3 protein at its centre. Although maize will have an analogous protein, it holds little biological meaning in the context of N responses as it is likely to bind to a very large number of proteins. Two components are associated with chloroplast thioredoxin and beta-glucosidases, but their role in this context is unclear. This analysis has provided some clues to the protein-level processes taking place in the response to low-N, especially the role of certain TFs, but the picture is far from complete.

Observation of the Study 1 dataset showed only nine of Ensembl mapped genes were present in IPA. The Table S4 listed these with their functional description. Several of these clustered into the network in Figure 3B. This network was held together by UBC (ubiquitin) [40], which is not very informative. The grey-coloured nodes represent the input genes. However, it showed that two of the DE genes interact with calmodulin, suggesting a role for calcium. Two of the input genes (UBE2H and CYP46A1) appeared to be regulated by TFs (a Ring Finger, RNF186 and a nuclear receptor RORA) [41,42,43,44], but it will be hard to discover the equivalent maize genes which are associated with the regulation of N metabolism. It indicated that there was various protein, protein interactions among the Study 1 gene products, but these networks are still too sparse to be of use. Therefore, it is worth using network inference techniques to see if any of these TFs can be linked to sets of genes known to be involved in responses to low N input.

### 3.3. Identification of N-Responsive Genes Using Artificial Neural Network Analysis

**Figure 4:**
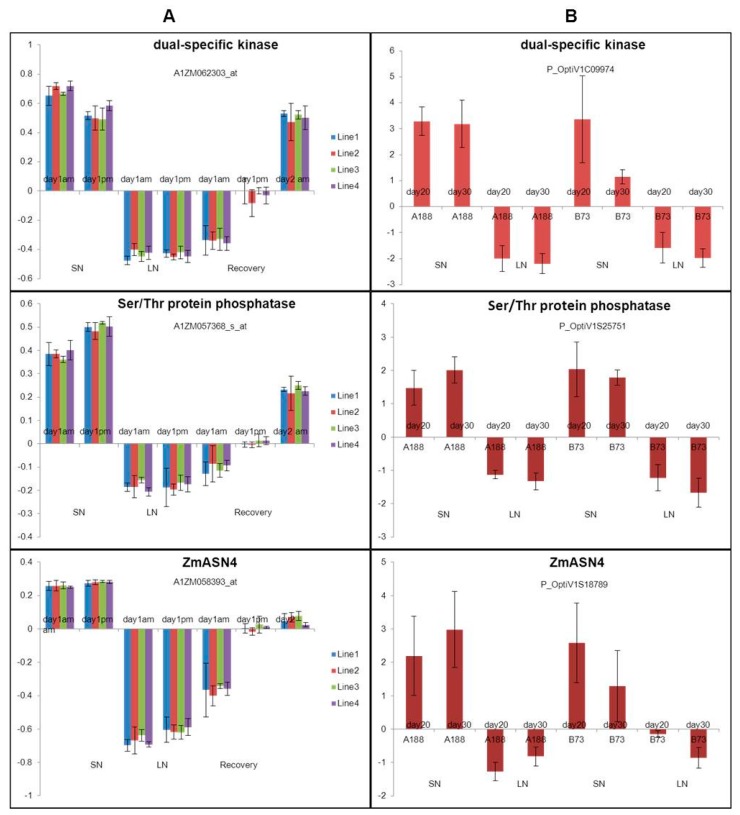
Expression patterns of three common genes selected from artificial neural network (ANN) across chips comparison. (**A**) Gene expression date from Study 1 [24], (**B**) Gene expression date from Study 2 [25]. Note: LN: low nitrogen; SN: sufficient nitrogen; Recovery: recovery from low N to SN.

ANN analysis of the transcription profiles from two different microarray datasets (Study 1 and Study 2) was focused on identifying the common genes from both studies associated with N use. The top-ranking 99 transcripts in each dataset were selected from each study to construct the network based on their mean square error for prediction of the level of N use efficiency. This approach studied the interactions between these and identified the strongest. The hubs derived from this approach are potentially the most influential genes in N use efficiency system. There were three top-ranking common genes: a dual-specific kinase, Ser/Thr phosphatase and ZmASN4 (with differential expression on both chips). These three genes were all on the list of Top 20 common down-regulated genes, and showed similar expression patterns (Table 5 and Figure 4).

**Figure 5:**
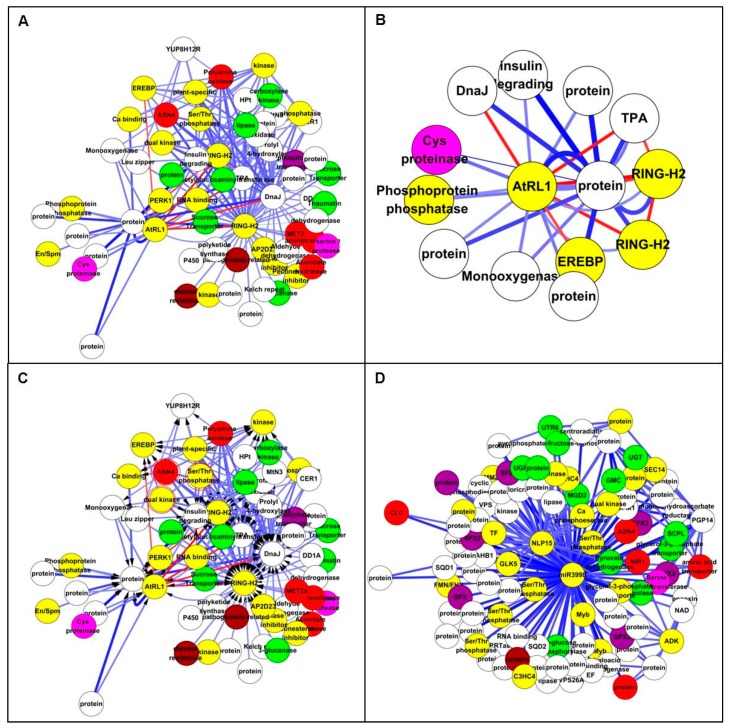
Top 200 interactions from all genes by ANN analysis. (**A**) All genes network with Top 200 interactions in Study 1. Nodes are coloured according to gene function: amino acid related in pink; carbon related in green; nitrogen related in red; phosphate related in purple; regulator and transcription factors (TFs) in yellow; stress related in brown; and others in white. (**B**) Extracted subnetwork in Study 1. AtRL1 interacted with other TFs such as RING-H2, EREBP and cysteine proteinase. (**C**) All genes network with Top 200 interactions. The nodes highlighted by red circle represent the three common genes: ZmASN4, dual specific kinase and Ser/Thr protein phosphatase. (**D**) Extracted sub-network with two central nodes: NLP15 and miR-399b. NLP15 and GLK5 were significantly down-regulated TFs under low N treatment.

In the Study 1 set, the Top 200 interactions included 65 transcripts (Figure 5A and Table S5), of which 32 showed differential expression. The top up-regulated TF on Affymetrix chip, AtRL1, is a member of a small subfamily of *Arabidopsis* RADIALIS-LIKE genes. AtRL1 was adjacent to 14 genes including cysteine proteinase, RING-H2 and EBERF (Figure 5B). In the Study 2 set, the Top 200 interactions included 99 transcripts (Figure 5C,D and Table S6), of which 91 probes were down-regulated and four were up-regulated. MiRNA-399b had a high vertex degree, interacting with 97 other nodes in inferred network, suggesting that it is an important regulator associated with N metabolism. It has been also proved to be a key regulator in systemic signalling, especially in Pi starvation [45]. Another high-degree node was ZmNLP15, which has been shown to play an important role in N regulation of nodule organogenesis [4], and recently it has been reported to be a central gene in nitrate signalling [11]. Two N-related genes were identified, one is a chloride channel-like (CLC) protein that is an anion channel and plays a key role in nitrate accumulation in plant vacuoles [46] and the second is NIR1, which plays a role in a nitrogen-regulated signalling pathway [47]. Interestingly, all of the node interactions were negative, indicating they were inhibited under low N condition (Figure 5C,D).

### 3.4. Identification of N-Responsive Transcription Factors Using Artificial Neural Network Analysis

**Figure 6:**
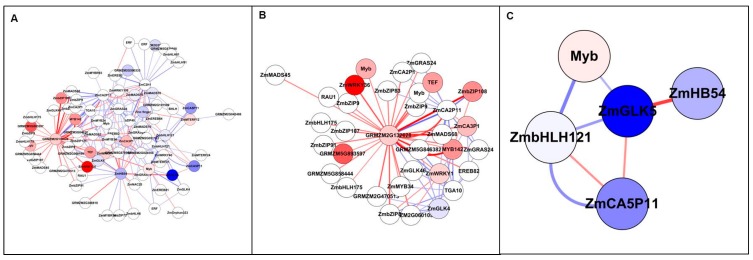
Top 200 interactions from TFs by ANN analysis (Study 1). All nodes are coloured based on fold change value (FC > 2): blue represents down-regulated, while red represents up-regulated. Edge with red colour indicates a positive interaction (stimulation), while edge with blue colour indicates a negative interaction (inhibition). The edge thickness based on *Z*-score value. (**A**) All TFs network with Top 200 interactions. Eighty-one TFs included 11 up-regulated and 13 down-regulated TFs. (**B**) Extracted subnetwork with GRMZM2G132628 as core hub surrounded with 30 positive interactions. (**C**) Extracted subnetwork with ZmGLK5 connected with four differentially expressed genes: ZmHB54, ZmbHLH121 and two TFs (GRMZM5G825312 and GRMZM5G868618).

All the TFs from both studies, 1626 and 2223 in Studies 1 and 2 respectively were selected, and their expression profiles subjected to ANN analysis. After the first stage ANN screening, the Top 99 TFs were selected based on mean square error, and then submitted to inferred interaction analysis. The overall predictive performance of the models was assessed based on the difference between the actual and predicted correlation, coefficient of each pair of 99 TFs. The number of interactions drastically decreased from 10,000 to 200 in terms of *Z*-score. This clearly showed that 24 out of Top 99 TFs were differentially expressed, while only five from the Study 2 dataset were differentially expressed (Figure 6 and Table S7).

In Study 1, there were 13 down-regulated and 11 up-regulated TFs as a result of N stress (Table S7). Figure 6 showed GRMZM2G132628 was found to have the highest positive interaction based on the highest vertex degree and stress, with 30 positive interactions (stimulation) with other TFs and three negative interactions (inhibition). This TF is homologous with rice LOC_Os06g50860 which is known to act on carbon-nitrogen balance. Interestingly, it appears as a key feature within the whole network, interacting strongly with high differentially expressed ZmbZIP108, MYB142, and ZmWRKY36 which are involved in signalling and responses to abiotic/biotic stress, possibly caused by low N stress. Opposingly the strongest interaction was identified between GRMZMG132628 with ZmCA2P11 and ZmGLK4, which corresponds to CCAAT-binding complex and cell-type differentiation processes in C4 plants, respectively. Moreover, some key nodes were identified showing down-regulation, with highly down regulated ZmGLK5, which belongs to the G2-like family and playing a specific role for transcriptional regulation and photosynthetic development in C4 plant differentiation. It is also adjacent to HB54, bHLH121, a MYB and CA5P11 (Figure 6C). 

**Figure 7:**
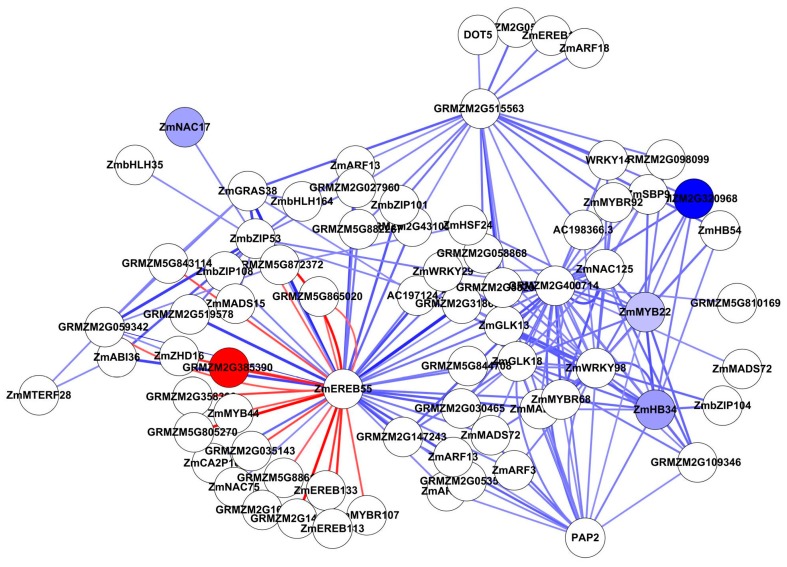
Top 200 interactions from TFs by ANN analysis (Study 2). All nodes are coloured by expression fold change value (FC > 2), blue represents down-regulated, while red represents up-regulated. Edge with red colour indicates a positive interaction (stimulation), while edge with blue colour indicates a negative interaction (inhibition). The edge thickness based on *Z*-score value.

In Study 2, the Top 200 interactions included 76 TFs, of which only one was up-regulated and four were down-regulated (Figure 7 and Table S8). The most interacting TF is ZmEREB55, associated with specific DNA-binding activity, but it was not differentially expressed. In addition, GRMZM2G400714, ZmWRKY98 and GRMZM2G515563 had high vertex degrees. ZmWRKY98 is involved in ABA biosynthesis and ABA-dependent responses to abiotic stress in vegetative tissue [48]. The other two genes have unknown function.

### 3.5. Analysis of Genome-Scale Networks—Subnetworks from All Genes Input

The Affymetrix array dataset including multiple N treatments, maize lines and transcripts, was used for Genome-Scale networks analysis. The 90 array expression profiles included 84,286 probes, which were normalized with the Robust Multiple-chip Analysis (RMA) algorithm by RMAExpress [23]. The inferred network included 41,841 genes and 8,631,218 links when a *z*-score threshold of 0.8, based co-expression network and spearman-based association methods, was used. To identify genes associated with N response, the Top 10 down/up-regulated genes were submitted (Table S9) as seed genes to exact sub-networks (Tables S10 and S11). The sub-networks were visualized with the DeGNServer (Figures S1 and S2) and Cytoscape (Figures S3 and S4). The down-regulated sub-network contained 110 genes and 1611 links, of which 90 genes were differentially expressed (fold change over ±2) (Table S10), while the up-regulated sub-network had 110 genes and 455 links, of which 40 genes were differentially expressed (Table S11). 

Figure S3 shows the down-regulated seed genes associated with genes for a hydrophobic protein (protein folding and protein-small molecule interactions), asparagine synthetase (key intermediates in nitrogen metabolism and nitrogen transport), a putative SEC14 cytosolic factor, a Ser/Thr protein phosphatase, putative dual-specific kinase, protochlorophyllide reductase A, chloroplast precursor, etc. Using up-regulated genes, the sub-network (Figure S2) showed genes associated with N metabolism, asparaginase, AtRL1, glutathione S-transferase (GST 31), a putative gamma-glutamyl transpeptidase and a protein kinase.

### 3.6. Analysis of Genome-Scale Networks—Subnetworks from Differentially Expressed Gene Input

In a further investigation, only the DE genes were used as input to the DeGNServer. In these Affymetrix datasets, there were 1282 genes with a *z*-score threshold of 0.85 by value-based co-expression network method were differentially expressed. The inferred network had 130,856 links. Sub-networks were extracted using multiple seed genes associated with N metabolism or candidate genes from the ANN analysis (Tables S6 and S12–S24). 

**Figure 8:**
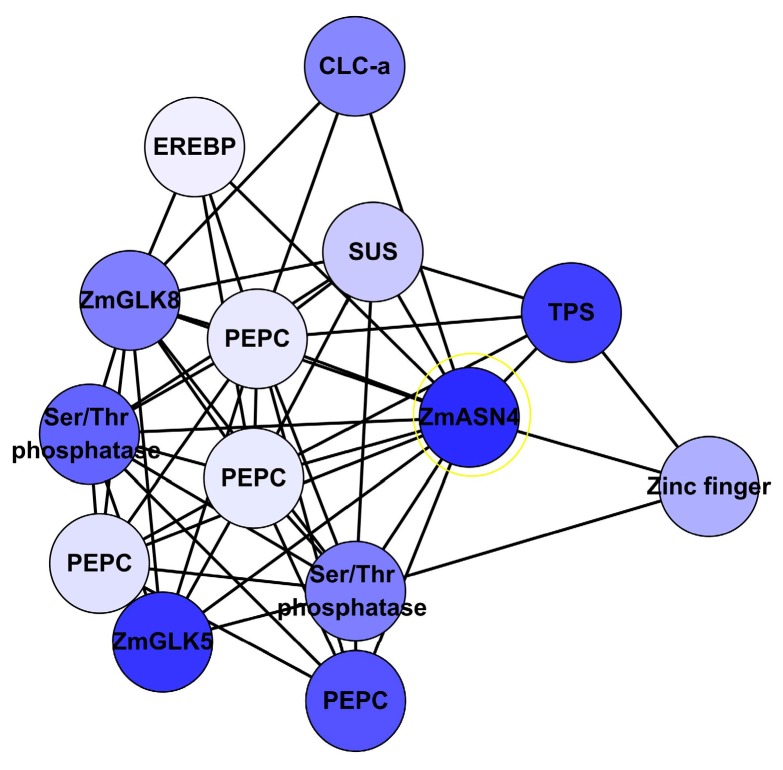
Sub-network of ZmASN4 interacted with its 13 neighbours. Note: CLC-a: chloride channel protein-a; EREBP: ethylene-responsive element binding protein; PEPC: phosphoenolpyruvate carboxylase; SUS: sucrose synthetase; TPS: terpene synthetase; ZmASN4: asparagine synthetase; ZmGLK5: G2-like 5 transcription factor; ZmGLK8: G2-like 8 transcription factor. Blue represents down-regulated gene.

Asparagine serves as an important major N storage and transport compound. Asparagine synthetase (ASN) gene acts as an important switching enzyme in the N metabolism [49]. ZmASN4 is one of the four distinct ASN mRNAs in maize, constituently expressed in leaf and root [50]. ZmASN4 is significantly down regulated under limiting N. In the network, ZmASN4 was adjacent to 130 genes including CLC-a, phosphoenolpyruvate carboxylase (PEPC), terpene synthase (TPS), and sucrose synthase (SUS). Regulation genes GLK5, GLK8, Ser/Thr phosphatase, Zinc finger, and EREBP were directly associated with ZmASN4 (Figure 8). Both GLK5 and GLK8 were identified on the ANN-TF analysis. PEPC plays important role in the C4 cycle, the CAM cycle and the citric acid cycle [51,52]. SUS is an important enzyme in starch and sucrose metabolism, and is regulated under abiotic stress [53,54,55]. Chloride channel (CLC) proteins display a variety of important physiological and cellular roles, which include regulation of pH, volume homeostasis, organic solute transport, cell migration, cell proliferation and differentiation. CLC-a was reported to mediate nitrate accumulation in *Arabidopsis* vacuoles [46]. However, there is no evidence for direct interaction between these genes, even in *Arabidopsis*.

**Figure 9:**
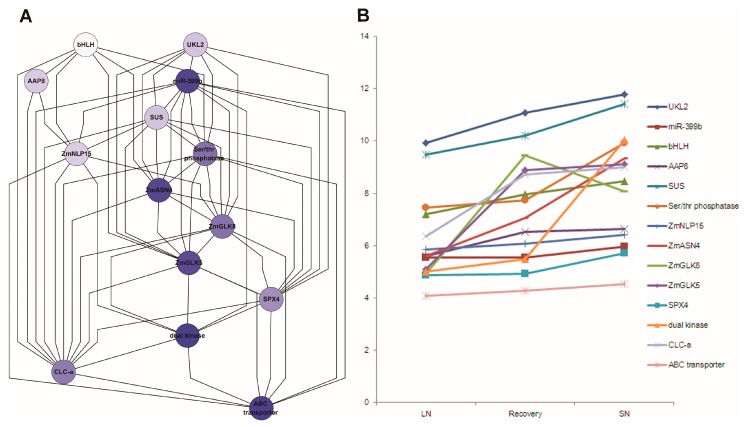
Subnetwork associated with N response genes (**A**); and their expression pattern (**B**) in maize leaves. 12A: Subnetwork with 14 N response genes was implemented in Cytoscape with hierarchic layout; 12B: Expression pattern of these N response genes under low N, sufficient N and N recovery. Note: LN: low nitrogen; SN: sufficient nitrogen; recovery: recovery from low N to SN; AAP8: Amino acid permease 8; bHLH: basic helix-loop-helix protein; CLC-a: chloride channel protein-a; SUS: sucrose synthetase; UKL2: Uridine kinase-like protein 2; ZmASN4: asparagine synthetase; ZmGLK5: G2-like 5 transcription factor; ZmGLK8: G2-like 8 transcription factor; ZmNLP15: nodule-inception like protein.

Fourteen co-related genes were integrated from the network analysis (Table S25), extracting their associations from network output, displayed by hieratic layout, and their expression profile level in Study 1 (Figure 9). These genes had a similar expression pattern under nitrogen treatments. ZmASN4, ZmNLP15 and ZmGLK5 were down-regulated under low N versus sufficient N treatment after germination 30 days.

### 3.7. qRT-PCR Analysis for Selected Candidate Genes

Five genes from the network analysis (e.g., ASN4, GLK5 dual kinase, and NLP15) and two N status genes (NR and NIR) were subjected to qRT-PCR analysis. ASN4 was significantly highly expressed in sufficient N in both maize lines B73 and B97. Between sufficient and low N, their fold changes were >20. NLP15, CLK5 and dual kinase showed higher expression in sufficient N (Table S26). These results well support the data shown in the networks and again indicate that these genes contribute to transcriptional responses to low N.

## 4. Discussion

This paper presents some of the first research on the combination of several network inference and analysis methods to identify novel genes associated with NUE. This has allowed integration of array datasets, and improving the predictive accuracy. From the study, we have discovered many candidate genes that are commonly induced by ammonium and nitrate. However, many specific genes have been uncovered from the two different N resources, which will be useful information for further dissecting the molecular mechanisms of N signalling pathways and their interactions with N responses and stress/defence responses.

One of the major factors influencing the accuracy of network analysis is their construction strategy/method. Given the diversity of proposed reverse engineering methods, it is recommended to combine different algorithms for a better understanding of the biology they represent [26]. For our work, we surveyed two networks of known interactions (BioGRID and IPA) and employed two inference algorithms (based on ANNs and co-expression-GNS). ANN based on mathematical modelling has previously shown strong capabilities, identifying the key regulators, especially transcription factors and patterns [32]. GNS a web-based Genome-scale gene network method could handle large expression data and provided a convenient way to extract genes and gene association, and to build up the potential sub-network based on gene expression and functional module [34]. A multi-layer feed-forward back-propagation ANN method was applied to selected TFs sub-network construction. ANN results give us a big hint of the TFs genes which have active performance response to N treatments. GNS method based on its powerful server could construct the genome-wide gene to gene interactions with large interactions. The high dimensional network requires a proper seed gene to search the sub-network. Usually applying key genes in a specific pathway and TFs selected from ANN as the seed gene could get a better reasonable sub-network. This also offers the advantage of explicitly capturing experimental provenance and increasing reproducibility. These findings provide some support for this interpretation.

The initial goal of this study was to compare published microarray datasets. This identified a set of 152 overlapping genes which are significantly either down- or up-regulated by N. Several classes of N-responsive genes have been highlighted, including those involved in a variety of metabolic and regulatory pathways. For example, the ABC transporter (GRMZM2G333224) is responsible for taking up nitrate or nitrite. The differentially expressing genes for amino acid and synthesis, including asparagine synthase, amino acid permease, serine acetyltransferase, threonine synthase, arginine decarboxylase, and anthranilate phosphoribosyltransferase show the involvement of primary N metabolism. Interestingly, UDP-galactosyltransferase related gene (GRMZM2G141320) has been found at the highest DE (FC > 6) under low N and sufficient N conditions. In common with the previous studies, we also observed UDP-galactosyltransferase related genes associated with lipid synthesis [56]. 

DE genes were mapped onto BioGRID and IPA, of these only nine were present in IPA. Most significantly, the node with the largest number of potential protein interactions encodes ZmMADS76. This is most remarkable for the fact that MADS-box TFs form multimers that recruit enzymes for histone modification and DNA methylation, i.e., epigenetic regulation. Previous studies showed that ASN gene family from *Arabidopsis* (ASN1, ASN2 and ASN3) seemed to play an important role on the nitrogen storage and transport compound used to allocate nitrogen resources. When ASN1 gene was overexpressed in *Arabidopsis*, transgenic plants with enhanced nitrogen status (seed protein contents) were obtained [57]. Interestingly, in our network, ZmASN4 seems to have strong and direct connections with CLC-a, PEPC, TPS, SUS and regulation genes GLK5, GLK8 Ser/Thr phosphatase, Zinc finger when using ZmASN4 as seed gene. Another candidate gene ZmGLK5 looked promising based on the network analysis. GLK family genes play key role in cell-type differentiation processes [38]. GLK1 and GLK2 are required for chloroplast development in *Arabidopsis* [58]. In *Arabidopsis*, NLP belongs to the NIN-like protein family associated with nitrate signalling [12]. It has been shown to bind to *Arabidopsis* Nitrate-response elements and is therefore an important observation. In C4 plants, NLP is a novel transcription factor family, which has a consensus motif conserved RWP-RK domain with the function in nitrogen-controlled development, and involved in nitrogen regulation of nodule organogenesis in lotus and legume plants [59]. ZmNLP15 could be a potential candidate gene for further studies. 

Based on BioGRID and ANN analysis, bZIP108 was found to have the highest positive interaction with GRMZM2G132628 and strong interaction with other proteins that are required for the formation of a regulatory complex with their DNA-binding domains. Thus, bZIP shows enhanced expression and/or DNA-binding activity following induction by low N stress, and plays a role in gene activation. It will be interesting to explore how in vivo protein–DNA binding specificities are established in future. MiRNA399b controls inorganic phosphate (Pi) homeostasis in *Arabidopsis*, rice and barley [60,61,62]. Trevisan et al. [18] have reported that miRNA could represent an important biological component of NUE from their transcriptomic analysis. 

Based on these results, we predict several important regulatory roles for a dual kinase, ZmGLK5, bZIP108, CLC-a and miRNA399b. They are obvious targets for developing maize mutants with improved NUE. This work has highlighted the advantages of data integration and advanced network inference analysis techniques, which, in principle, can be applied to many other plant and animal systems. 

## Figures and Tables

**Table 1:** Details of two maize microarray studies.

	Study 1	Study 2
Authors	Yang et al., 2011 [24]	Schluter et al., 2012 [25]
Aim	Identify biomarkers of Nitrogen status	Leaf responses to low Nitrogen
Platform	Affymetrix array	Agilent array
Plant varieties	4 commercial hybrids	Inbred A188 and B73
Growth conditions	Greenhouse	Growth chamber (14 h light + 10 h dark)
Sufficient N	20 mM NH_4_NO_3_	15 mM KNO_3_
Low N	2 mM NH_4_NO_3_	0.15 mM KNO_3_
Harvest	Plants sown so that all sample were harvested on same day. V6 leaves taken (at 21 days for sufficient N and 28 days for low N) plus following day for Line 4 and N-recovered plants. 3 replicates. 4 plants per replicate. Material harvested at 10 am and 11 pm on both days.	Material from 2 identical experiments combined. V5 and V6 leaves taken respectively at 20 and 30 days. 4 replicates. 1 plant from each experiment per replicate. Material harvested after 2 h of light.

N: Nitrogen.

**Table 2:** Integration of two sets of N fertilizer response microarray data via reference genome B73.

Microarray	Total Probes	Mapped Probes (Genes)	DE Probes (Genes) FC > 1.5	Down Regulated Genes	Up Regulated Genes	Conflicted Genes
Study 1	84,246	52,304 (31,958)	1282 (1088)			
Study 2	41,838	35,086 (27,278)	1009 (923)			
Common genes	□	(19,991)	165	136	16	13

**Table 3:** Summary of six genotypes gene expression pattern under low versus sufficient nitrogen treatments.

Microarray	Genotype	Differentially Regulated Transcripts					
		Down	Up	Total	Retrieved Down	Retrieved Up	Conflicted
Study 1	Line 1	1268	2391	3659			
	Line 2	1194	827	2021			
	Line 3	914	1529	2443			
	Line 4	1329	1093	2422			
	Lines 1–4	713	569	1282	596	492	
Study 2	A188 (D30)	1506	675	2181			
	B73 (D30)	1608	1474	3082			
	A188 and B73 (D30)	703	306	1009	639	284	
	Lines 1 and 3, and A188				184	39	30
	Lines 1 and 3, and B73				158	66	70
	Lines 2 and 4, and A188				240	25	22
	Lines 2 and 4, and B73				203	40	56
Studies 1 and 2	Lines 1–4, A188, and B73				136	16	13

**Table 4:** Top common down-regulated and up-regulated genes under low N versus sufficient N treatments (Lines 1–4, A188 and B73).

MaizeGDB Gene	Affy ID	Agi ID	FC	Gene Description
GRMZM2G141320	A1ZM005708_at	P_OptiV1C15662	−97.7	UDP-galactosyltransferase
GRMZM2G333224	A1ZM067520_at	P_OptiV1S29808	−64.4	abc transporter
GRMZM2G018820	A1ZM001292_s_at	P_OptiV1C11631	−45.6	GDE1
GRMZM2G016370	A1ZM060059_at	P_OptiV1C16142	−39.1	ZmGLK5
GRMZM2G100454	A1ZM062303_at	P_OptiV1C09974	−35.3	dual-specific kinase
GRMZM2G373607	A1ZM045918_at	P_OptiV1S32106	−32.2	SCPL
GRMZM2G086179	A1ZM005813_s_at	P_OptiV1N40557	−28.6	
zma-MIR399b	A1ZM015359_at	P_OptiV1N41856	−26.4	zma-MIR399b
GRMZM2G176562	A1ZM019400_at	P_OptiV1S17969	−24.9	SQD2
GRMZM2G060311	A1ZM015149_at	P_OptiV1S18616	−24.6	hydrophobic protein OSR8-like
GRMZM2G152447	A1ZM002117_at	P_OptiV1C10994	−22.0	Purple acid phosphatase 1
GRMZM2G526727	A1ZM055955_at	P_OptiV1C13366	−21.0	Arginine decarboxylase
GRMZM5G805389	A1ZM017973_at	P_OptiV1C03719	−19.6	SPX domain
GRMZM5G853702	A1ZM038244_at	P_OptiV1N38765	−19.2	bowman-birk type trypsin inhibitor precursor
GRMZM2G043565	A1ZM013564_at	P_OptiV1N41328	−18.6	
GRMZM2G035579	A1ZM024880_at	P_OptiV1N39672	−16.6	spx domain-containing protein
GRMZM2G078633	A1ZM058393_at	P_OptiV1C00703	−16.4	asparagine synthase (ZmASN4)
GRMZM2G344654	A1ZM003814_s_at	P_OptiV1C00507	−16.1	
GRMZM2G049541	A1ZM055541_at	P_OptiV1C04552	−16	phosphoenolpyruvate carboxylase kinase
GRMZM2G069694	A1ZM006239_at	P_OptiV1C03616	−15.8	Plant specific
GRMZM5G816348	A1ZM062346_at	P_OptiV1C03692	10.3	oligopeptide transporter
GRMZM2G355752	A1ZM078046_x_at	P_OptiV1S20690	7.6	early light-induced protein
GRMZM2G087507	A1ZM056359_at	P_OptiV1C01116	6.3	probable nad h-dependent oxidoreductase
GRMZM2G177098	A1ZM000024_a_at	P_OptiV1C14892	5.1	terpene synthase
GRMZM2G330635	A1ZM011141_at	P_OptiV1S29121	4.7	glutathione s-transferase
GRMZM2G455582	A1ZM069754_at	P_OptiV1S29347	4.2	pentatricopeptide repeat
AC185430.3	A1ZM023429_at	P_OptiV1N39003	3.7	Rho guanyl-nucleotide
GRMZM5G812407	A1ZM055479_at	P_OptiV1C10233	3.6	ZmC3H51
GRMZM5G857944	A1ZM003474_a_at	P_OptiV1C16320	3.5	ZmCA2P13
GRMZM2G367907	A1ZM003724_s_at	P_OptiV1N39816	3.3	IMP dehydrogenase
GRMZM2G032807	A1ZM055038_s_at	P_OptiV1S28735	3.1	heavy metal-associated domain
GRMZM2G359664	A1ZM027751_at	P_OptiV1S21353	3.1	pollen-specific kinase
GRMZM2G016323	A1ZM046170_s_at	P_OptiV1N38397	3.0	ubiquitin carboxyl-terminal hydrolase
GRMZM2G177169	A1ZM005058_at	P_OptiV1S24357	2.4	pentatricopeptide repeat
GRMZM2G440349	A1ZM041153_at	P_OptiV1S27652	2.4	pentatricopeptide repeat
GRMZM2G462625	A1ZM012815_at	P_OptiV1S23216	2.3	pentatricopeptide repeat

FC (fold change) > ±2, *p*-value < 0.05.

**Table 5:** Three common genes of ANNs-all transcripts across chips comparison.

MaizeGDB Gene ID	Affy ID	Agi ID	log_2_FC (Affy)	log_2_FC (Agi)	Gene Description
GRMZM2G100454	A1ZM062303_at	P_OptiV1C09974	−64.9	−18.9	Putative dual-specific kinase
GRMZM2G134054	A1ZM057368_s_at	P_OptiV1S25751	−11.6	−10.6	Ser/Thr protein phosphatase
GRMZM2G078633	A1ZM058393_at	P_OptiV1C00703	−34.1	−7.9	ZmASN4

FC > ±2, *p*-value < 0.05.

## Supplementary Materials

The following are available online at http://www.mdpi.com/2073-4425/9/3/151/s1. Figure S1: Subnetwork of GSN with Top 300 interactions constructed by Top 10 down-regulated genes (Study 1), Figure S2: Subnetwork of GSN with Top 300 interactions constructed by Top 10 up-regulated genes (Study 1), Figure S3: GSN subnetwork constructed by Top 10 down-regulated genes (Study 1), Figure S4: GSN subnetwork constructed by Top 10 up-regulated genes (Study 1), Table S1: Common differential expression transcripts compared limiting versus high nitrogen treatment via crossing chips comparison, Table S2: List of the core N response genes in Study 1 mapped to *Arabidopsis* genome, Table S3: The known *Arabidopsis* networks which were retrieved by the core N response biomarkers in Study 1, Table S4: List of transcripts with functional description from IPA analysis, Table S5: Top 200 interactions (65 nodes) of ANN (Study 1) sorted by vertical degree, Table S6: Top 200 interactions (99 nodes) of ANN (Study 2) sorted by vertical degree, Table S7: Top 200 interactions (81 nodes) of ANN-TFs (Study 1) sorted by vertical degree, Table S8: Top 200 interactions (76 nodes) of ANN-TFs (Study 2) sorted by vertical degree, Table S9: Top 10 down/up regulated genes under low N vs sufficient N in Study 1, Table S10: Sub-network (Study 1) which was built up by using Top 10 down regulated genes as seed genes, Table S11: Sub-network (Study 1) which was built up by using Top 10 up regulated genes as seed genes, Table S12: Sub-network which was built up by using ZmASN4 as seed gene, Table S13: Sub-network which was built up by using Asparaginase as seed gene, Table S14: Sub-network which was built up by using CLC-a as seed gene, Table S15: Sub-network which was built up by using as seed gene, Table S16: Sub-network which was built up by using PEPC as seed gene, Table S16: Sub-network which was built up by using PEPC as seed gene, Table S17: Sub-network which was built up by using SUS as seed gene, Table S18: Sub-network which was built up by using Ser/Thr phosphatase as seed gene, Table S19: Sub-network which was built up by using dual-specific kinase as seed gene, Table S20: Sub-network which was built up by using Myb as seed gene, Table S21: Sub-network which was built up by using Myb-like as seed gene, Table S22: Sub-network which was built up by using RWP-RK as seed gene, Table S23: Sub-network which was built up by using mir-399b as seed gene, Table S24: Sub-network which was built up by using AtRL1 as seed gene, Table S25: 14 co-related N response genes which were integrated from genome scale network analysis, Table S26: Relative real-time expression of ZmASN4, GS2, NR, NIR, ZmGLK5, dual kinase and ZmNLP15.
