# Supplementary figures and images for: Analysis of Gene Regulatory Networks of Maize in Response to Nitrogen
Source: Genes (Basel). 2018 Mar 8;9(3):151. doi: 10.3390/genes9030151 (PMC5867872; doi:10.3390/genes9030151)

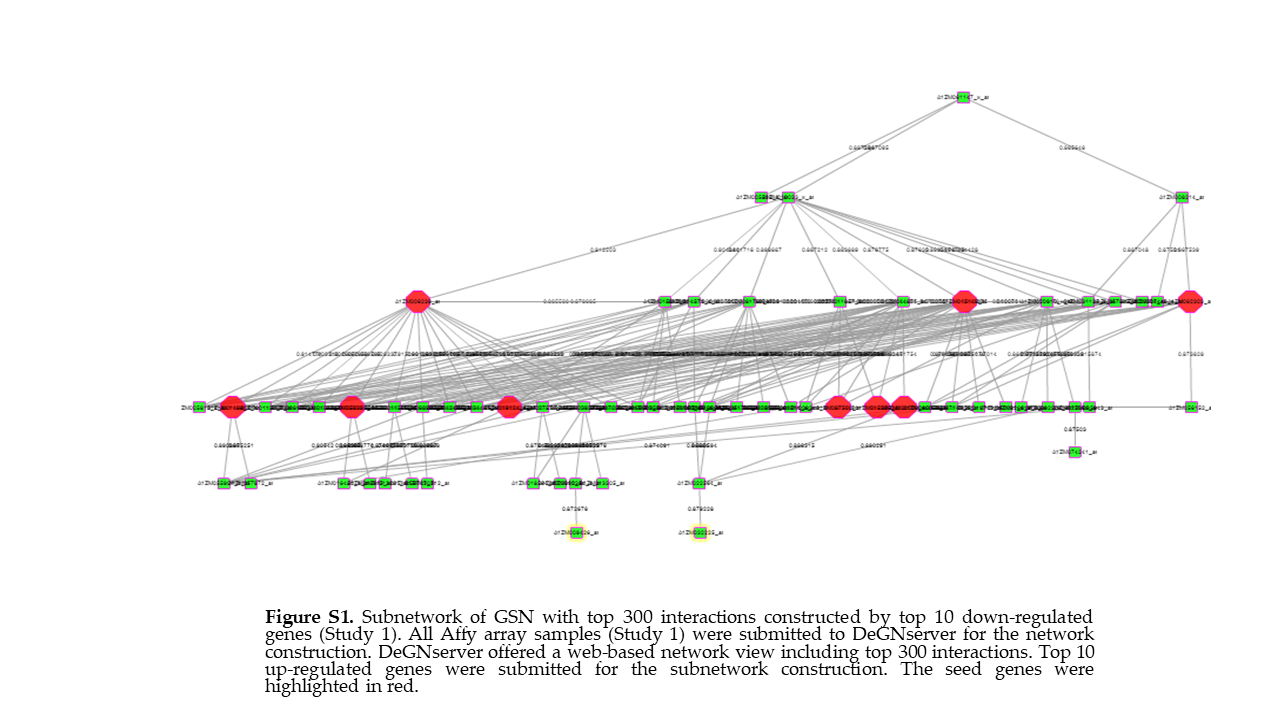

Supplement: Supplementary file 1 [file genes-09-00151-s001.zip › FigureS1.tif]

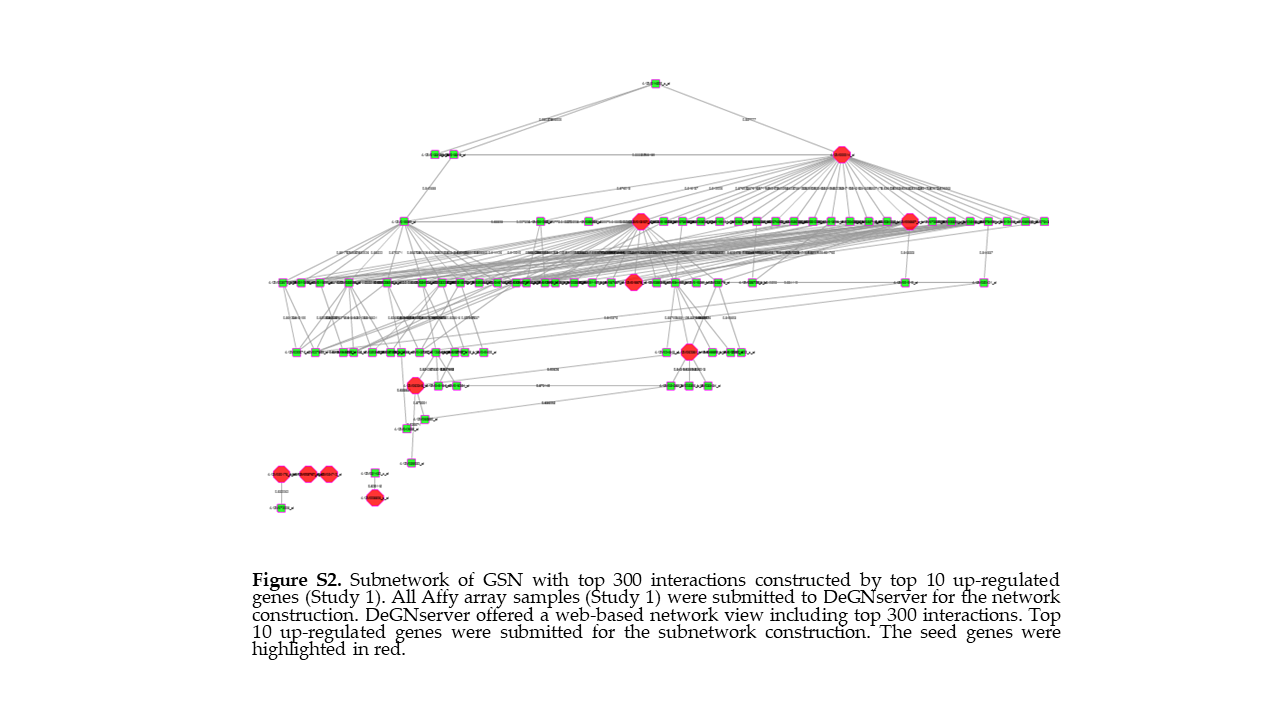

Supplement: Supplementary file 1 [file genes-09-00151-s001.zip › FigureS2.tif]

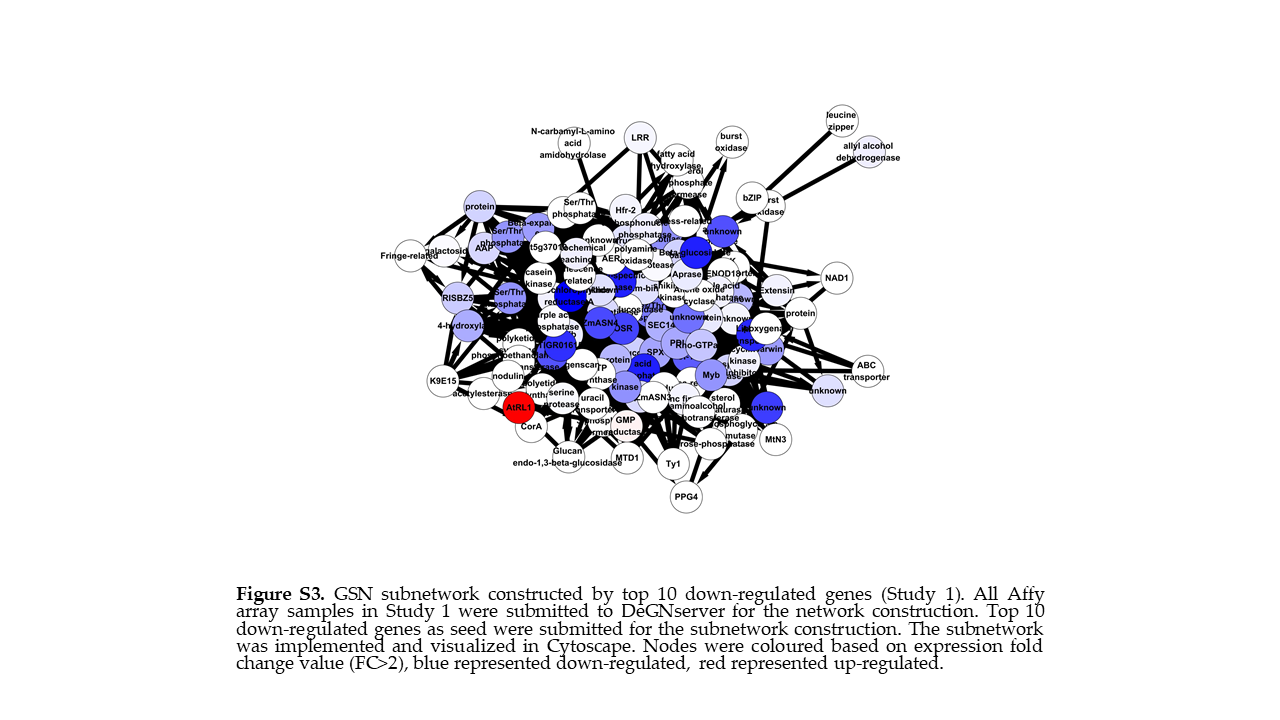

Supplement: Supplementary file 1 [file genes-09-00151-s001.zip › FigureS3.tif]

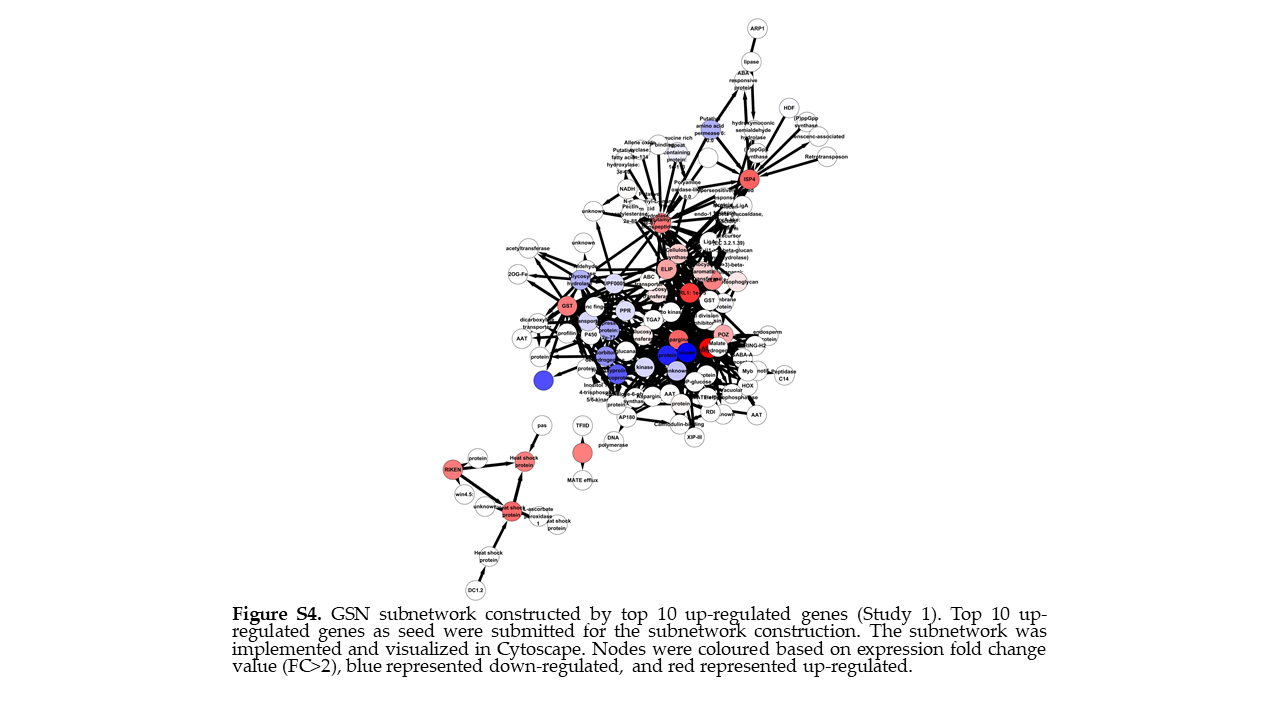

Supplement: Supplementary file 1 [file genes-09-00151-s001.zip › FigureS4.tif]
